# Case Report: Four cases of cardiac sarcoidosis in patients with inherited cardiomyopathy—a phenotypic overlap, co-existence of two rare cardiomyopathies or a second-hit disease

**DOI:** 10.3389/fcvm.2023.1328802

**Published:** 2023-12-19

**Authors:** Hans Ebbinghaus, Laura Ueberham, Daniela Husser-Bollmann, Andreas Bollmann, Ingo Paetsch, Cosima Jahnke, Ulrich Laufs, Borislav Dinov

**Affiliations:** ^1^ Department for Electrophysiology, Heart Center of Leipzig, Leipzig, Germany; ^2^ Klinik und Poliklinik für Kardiologie, Universitätsklinikum Leipzig, Leipzig, Germany; ^3^ Department of Cardiology, Medical University of Giessen, Giessen, Germany

**Keywords:** cardiac sarcoidosis, non-ischemic cardiomyopathy, familial cardiomyopathy, ventricular tachyarrhythmias, conduction disease

## Abstract

Cardiac sarcoidosis (CS), a rare condition characterized by non-caseating granulomas, can manifest with symptoms such as atrioventricular block and ventricular tachycardia (VT), as well as mimic inherited cardiomyopathies. A 48-year-old male presented with recurrent VT. The initial ^18^F-fluorodeoxyglucose positron emission tomography (^18^FDG-PET) scan showed uptake of the mediastinal lymph node. Cardiovascular magnetic resonance (CMR) demonstrated intramyocardial fibrosis. The follow-up ^18^FDG-PET scan revealed the presence of tracer uptake in the left ventricular (LV) septum, suggesting the likelihood of CS. Genetic testing identified a pathogenic *LMNA* variant. A 47-year-old female presented with complaints of palpitations and syncope. An Ajmaline provocation test confirmed Brugada syndrome (BrS). CMR revealed signs of cardiac inflammation. An endomyocardial biopsy (EMB) confirmed the diagnosis of cardiac sarcoidosis. Polymorphic VT was induced during an electrophysiological study, and an implantable cardioverter-defibrillator (ICD) was implanted. A 58-year-old woman presented with sustained VT with a prior diagnosis of hypertrophic cardiomyopathy (HCM). A genetic work-up identified the presence of a heterozygous *MYBC3* variant of unknown significance (VUS). CMR revealed late gadolinium enhancement (LGE), while the ^18^FDG-PET scan demonstrated LV tracer uptake. The immunosuppressive therapy was adjusted, and no further VTs were observed. A 28-year-old male athlete with right ventricular dilatation and syncope experienced a cardiac arrest during training. Genetic testing identified a pathogenic mutation in *PKP2*. The autopsy has confirmed the presence of ACM and a distinctive extracardiac sarcoidosis. Cardiac sarcoidosis and inherited cardiomyopathies may interact in several different ways, altering the clinical presentation. Overlapping pathologies are frequently overlooked. Delayed or incomplete diagnosis risks inadequate treatment. Thus, genetic testing and endomyocardial biopsies should be recommended to obtain a clear diagnosis.

## Background

The prevalence of cardiac sarcoidosis (CS) in patients with pulmonary sarcoidosis has been reported to be approximately 2%–7%, although autopsy studies have revealed myocardial granulomas in 20%–30% of cases, suggesting that the actual prevalence may be underestimated (1). Most probably, sarcoidosis has no single cause, and various exogenous and endogenous factors, including genetic predisposition, may play a role (2). The diagnosis remains challenging due to the non-specific nature of the symptoms that can include atrioventricular (AV) block, ventricular arrhythmias, heart failure, and syncope or even sudden cardiac death (1, 2). Furthermore, CS can present with features of dilated cardiomyopathy (DCM), hypertrophic cardiomyopathy (HCM), or arrhythmogenic cardiomyopathy (ACM), leading to misdiagnosis and delayed treatment (2–4). Potential genetic factors and the possibility of overlap syndromes between CS and inherited cardiomyopathies are not well-studied.

Here, we present four cases of patients diagnosed with CS and who were subsequently diagnosed with another inherited cardiomyopathy. Our purpose is to elucidate the complex interactions between both conditions influencing the symptoms, the course of the disease, and the outcomes.

## Case 1: sarcoidosis and *LMNA/C* DCM

**Figure 1:**
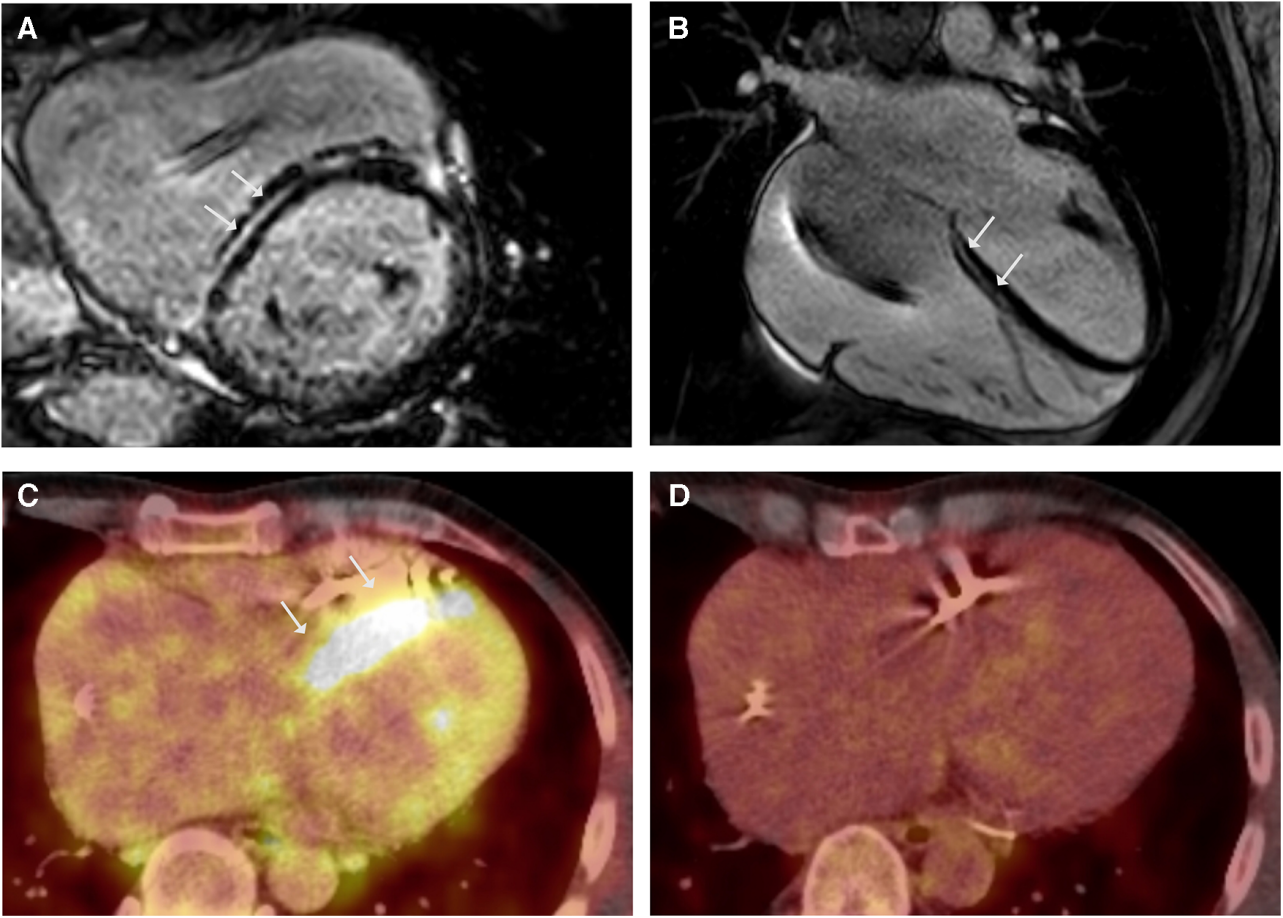
LGE CMR frames in short-axis (**A**) and 4-chamber (**B**)-views demonstrating striated midwall fibrosis of the septal LV wall (arrows). Ejection fraction was mildly impaired (LVEF 47%). (**C**) Axial-view of ^18^FDG-PET follow-up revealing tracer uptake in the LV Septum (arrows). (**D**) ^18^FDG-PET follow-up under immunosuppressive therapy with glucocorticoids (GC) showing no more tracer uptake indicating no cardiac inflammation under GC.

A 38-year-old male patient presented with persistent shortness of breath on exertion following an upper respiratory tract infection. Transthoracic echocardiography (TTE) revealed a discrete apical hypokinesia. Suspecting myocarditis, a cardiac magnetic resonance imaging (CMR) scan was performed, revealing reduced systolic left ventricular (LV) function (EF 44%) and evidence of focal inferolateral basal edema, consistent with myocardial inflammation. Coronary angiography ruled out coronary artery disease (CAD), and endomyocardial biopsies (EMB) were obtained excluding acute or chronic myocarditis. After innating an angiotensin-converting enzyme (ACE) inhibitor, the patient was subjectively symptom-free, with an improvement in the systolic LV function (EF 60%) during the 1-month follow-up. Three years later, the patient presented again with a third-degree atrioventricular block and a reduced LV function (EF 45%). As a result, a cardiac resynchronization therapy (CRT)-defibrillator was implanted. At 48 years old, he was admitted again due to recurrent ventricular tachycardia (VT). The device interrogation revealed clusters of non-sustained VT, a second-degree AV block, and frequent premature ventricular contractions (PVC). Due to the early onset of AV block and VT, CS was suspected, and the patient underwent ^18^F-fluorodeoxyglucose positron emission tomography (^18^FDG-PET), which revealed significant tracer uptake in multiple mediastinal lymph nodes but normal cardiac uptake. In combination with elevated serum levels of angiotensin-converting enzyme (105 U/L, reference range in U/L: 20–70), these findings were indicative for pulmonary sarcoidosis (5). A cardiovascular magnetic resonance (CMR) demonstrated a mildly impaired left ventricular ejection fraction (LVEF 47%) and striated intramyocardial fibrosis areas (Figures 1A,B), which was interpreted as pulmonary sarcoidosis with cardiac involvement in fibrotic, indolent stage. The symptomatic PVC from the LV outflow tract was successfully ablated, followed again by EMB. EMB was negative for cardiotropic viruses, and showed a low-grade lymphocytic inflammation without evidence of non-caseating granulomas consistent with DCM. However, CS could not be ruled out due to a potential sampling error. According to the synopsis of the findings, persistent lymphocytic inflammation, rapid progression, and young age led to the diagnosis of sarcoidosis with cardiac involvement in accordance with the most recent criteria of the Japanese Circulation Society (JCS), and glucocorticoid (GC) therapy was initiated (5). The ^18^FDG-PET follow-up scan after 6 months revealed tracer uptake in the LV septum (Figure 1C) and mediastinal lymph nodes, indicating active sarcoidosis despite the GC treatment. Thus, the prednisolone dose was increased. The subsequent ^18^FDG-PET scan showed a decreased lymph node inflammation and no cardiac inflammation (Figure 1D). The positive response to immunosuppressive therapy supported the diagnosis of sarcoidosis. Nonetheless, genetic testing was performed due to a positive family history of DCM (mother and uncle on the maternal side), revealing a heterozygous pathogenic variant (Arg321Ter) in the *LMNA* gene.

## Case 2: sarcoidosis and Brugada syndrome

**Figure 2:**
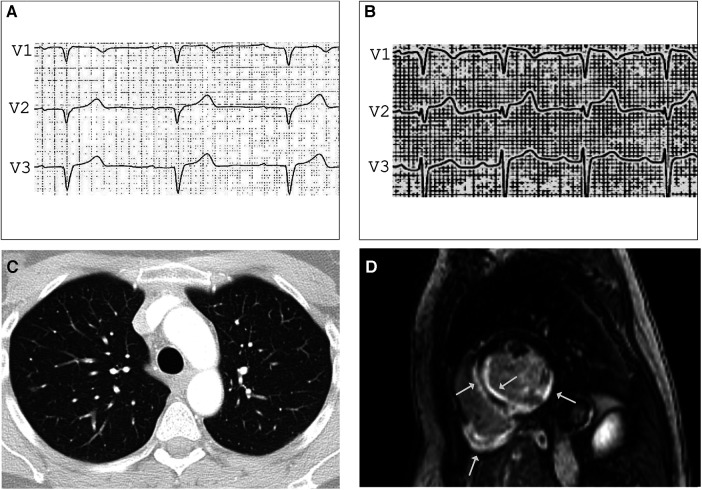
ECG before (**A**) and after (**B**) Ajmalin provocation test unmasking coved-type ST segment elevation in V1 suggesting BrS. (**C**) Contrast-enhanced CT showing small, irregular foci in both lungs compatible with pulmonary sarcoidosis stage 3. LGE CMR frame in short-axis - view (**D**) revealing subendocardial to intramyocardial LGE in the basal septal and inferolateral LV wall and the entire right ventricular posterior wall (arrows), LVEF was mildly impaired (41%) with normal-sized heart chambers.

A 47-year-old female medical assistant was referred to our department due to recurrent palpitations, two prior unexplained presyncope, and suspicion of ventricular septal defect. There was no family history of sudden cardiac death (SCD), cardiomyopathy, or sarcoidosis. The echocardiography showed basal thinning of the interventricular septum but no septal defect. The ECG showed left anterior fascicular block, frequent PVCs, and normal QTc. The Ajmaline provocation test unmasked a coved-type ST segment elevation in V1 with inverted T-waves (Figures 2A,B), suggesting the Brugada syndrome. To exclude the presence of structural heart disease, a CMR was performed 2 days after the Ajmaline provocation test was conducted, revealing an impaired LVEF of 41% with normal-sized heart chambers. Surprisingly, the T2 mapping demonstrated edema at the posterior wall of the right ventricle, as well as at the septal, anterior, and posterior walls of the left ventricle (LV). Furthermore, late gadolinium enhancement (LGE) was observed in the subendocardial to intramyocardial regions of the basal septal and inferolateral LV wall, as well as the entire posterior wall of the right ventricle (Figure 2D). A contrast-enhanced CT showed small, irregular parenchymal foci dispersed in both lungs without evidence of mediastinal lymphadenopathy or fibrosis (Figure 2C). Due to the signs of inflammation shown in the CMR and CT scans, suggesting stage 3 pulmonary sarcoidosis, we performed an EMB, which was positive for epithelioid granuloma with multinucleated cells, confirming the diagnosis of CS. Considering the impaired LVEF, the presence of LGE, and two previous episodes of presyncope, an electrophysiological study for risk stratification was performed that resulted in the induction of polymorphic VT, and a dual chamber implantable cardioverter-defibrillator (ICD) was implanted. During the 6-month follow-up, the device interrogation revealed episodes of polymorphic non-sustained VT and spontaneously terminating ventricular fibrillation (VF).

## Case 3: sarcoidosis and HCM

**Figure 3:**
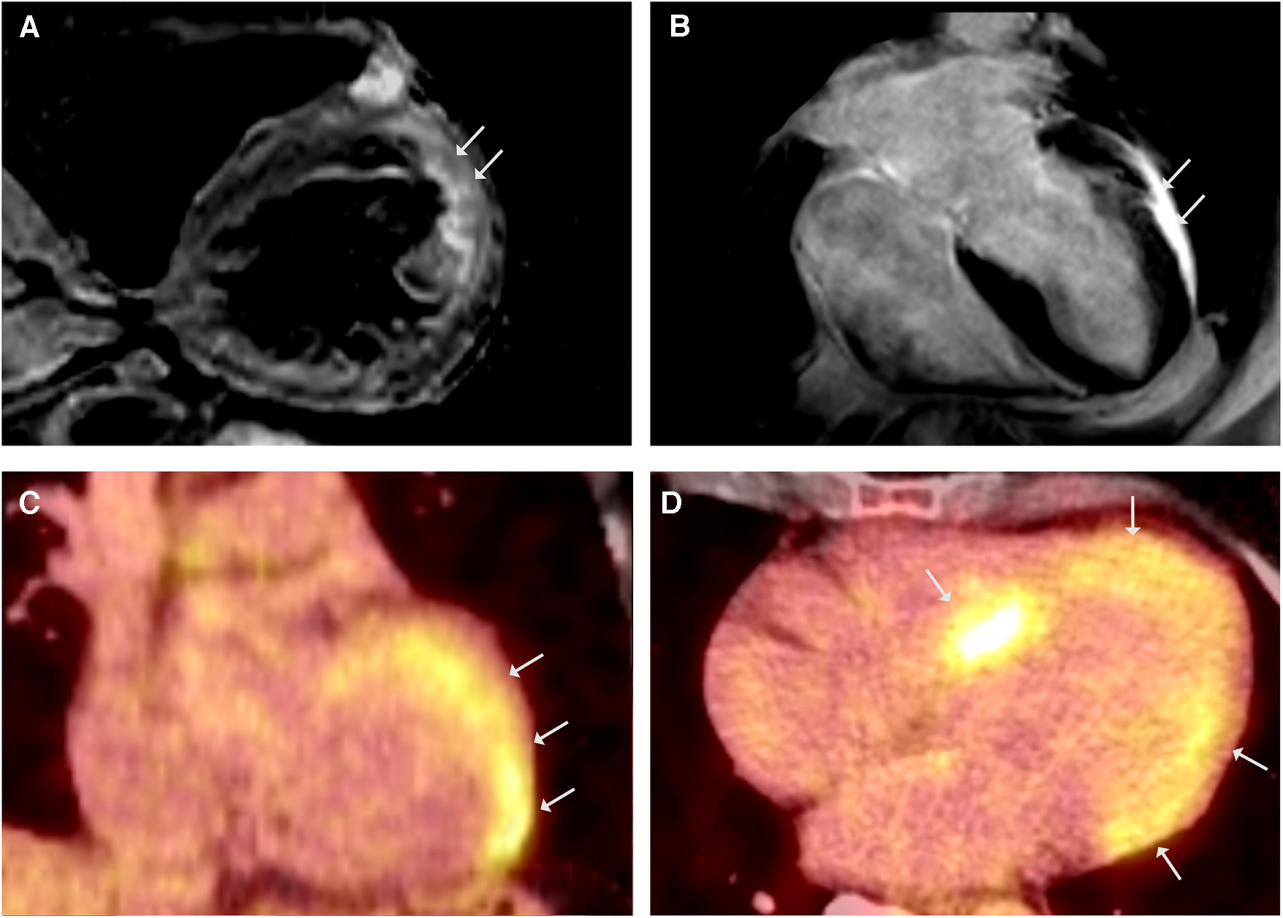
CMR showed simultaneously occurring edematous and fibrosing processes. (**A**) CMR, T2-weighted black blood imaging in short - axis view demonstrating regional edema of the LV anterolateral wall. (**B**) LGE CMR frame in 4-chamber view showing LV hypertrophy and anterolateral LGE, LVEF was mildly reduced (43%). ^18^FDG-PET coronal (**C**) and axial (**D**) views revealing intense FDG uptake in septal and lateral LV.

A 58-year-old woman was referred to our department due to sustained VT with the diagnosis of HCM in a previous CMR scan. The genetic work-up identified a heterozygous *MYBPC3* variant (p.Arg1268Trp) as a variant of unknown significance (VUS). Her history for familial cardiomyopathies or SCD was negative. The ECG showed a first-degree AV block, right bundle-branch block, and left anterior block. Repeated CMR scans confirmed the presence of concentric LV hypertrophy, reduced LVEF (43%), and anterolateral LGE (Figure 3B). Notably, T2 mapping demonstrated regional edema of the LV anterolateral wall (Figure 3A) and raised the suspicion of CS in (sub)acute inflammatory stage. The ^18^FDG-PET scan showed intense LV septal and lateral FDG uptake (Figures 3C,D), without evidences of extracardiac involvement. The coronary angiography ruled out relevant stenosis, and EMB were taken from the interventricular septum. In the molecular pathological examination, non-caseating granulomas were absent; however, CS was deemed very probable based on the imaging criteria established by Vita et al. (5, 6), and the diagnosis of isolated cardiac sarcoidosis was made according to the JCS criteria. The patient refused ICD implantation and was discharged on GC therapy. Three months later, the ^18^FDG-PET scan showed increased tracer uptake in the apex as well as decreased enhancement in the septum, and the immunosuppressive therapy was augmented with methotrexate. No further VTs were observed in the patient during the follow-up period.

## Case 4: sarcoidosis and ACM

**Figure 4:**
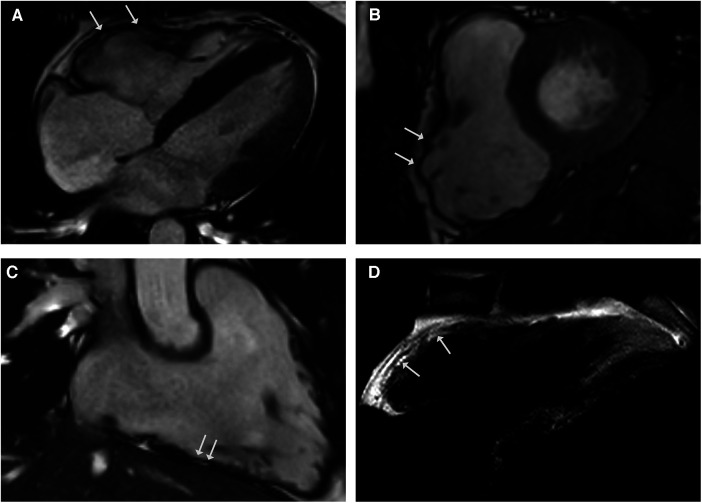
CMR cine images in 4-chamber (**A**) and short-axis - views (**B**) revealing right ventricular dilatation with bulging (arrows) and regional dyssynchrony in end-systolic phase. Right ventricular systolic function was mildly reduced (RV EF 42%). CMR 3-chamber view of the RV (**C**) demonstrating fatty spots. (**D**) T1-weighted black blood spin CMR showing fatty replacement of the RV free wall.

A 28-year-old male athlete with a history of exercise-induced syncope presented at our clinic. He reported palpitations and loss of consciousness during intensive physical activity as an American football player. His family history was negative for sudden cardiac death. The clinical examination, blood testing, and 12-lead ECG were unremarkable. The transthoracic echocardiogram showed right ventricular enlargement without dyskinesia. Exercise testing and signal-averaged ECG were normal. The CMR revealed a dilated right ventricle with regional dyssynchrony and moderately decreased right ventricular ejection fraction of 42% (Figures 4A–D). The RV end-diastolic volume index was measured at 130 ml/m^2^. Despite the strong recommendation against competitive sports, he suffered a cardiac arrest during training, with ECG showing ventricular fibrillation. Immediate defibrillation failed, and he was transferred to our institution under ongoing cardiopulmonary resuscitation. Sinus rhythm was restored post-ECMO, but the patient died due to cerebral edema. The postmortem genetic testing identified a novel pathogenic mutation in plakophilin-2 (*PKP2*), leading to premature truncation (NM_004572.3 c.1540_1543delAAAC). The autopsy confirmed ACM, and extracardiac sarcoidosis was found in multiple organs including the lungs, pleura, spleen, liver, lymph nodes, and pelvis. Notably, there was no evidence of cardiac involvement in the samples examined.

## Discussion

This case series reveals the complex interactions between CS and inherited cardiomyopathies ranging from simple coexistence or phenocopying to overlap cardiomyopathy with distinct clinical presentation and outcomes.

The diagnosis of cardiac sarcoidosis was made according to the most recent guidelines by the JCS for diagnosing cardiac sarcoidosis (5). We employed these criteria due to the diagnostic challenges associated with isolated cardiac sarcoidosis, primarily arising from potential sampling errors in endomyocardial biopsy. Notably, Kandolin et al. (7) found that at least two-thirds of patients remain undiagnosed after a single endomyocardial biopsy session. In contrast to the criteria set by the Heart Rhythm Society (HRS), the criteria established by the Japanese Circulation Society allow for diagnosis even in the absence of histological evidence of cardiac or extracardiac non-caseating granulomas (5, 8). Sato et al. (9) have substantiated the reliability of the updated JCS diagnostic criteria, using a multimodal imaging approach. However, the lack of histological evidence remains a limitation in the context of overlap cardiomyopathies.

*LMNA/C*-related DCM and CS can conceivably coexist without influencing each other. Due to the similar clinical presentation, they cannot be reliably distinguished from one another. Both CS and *LMNA/C*-associated DCM usually present at early to mid-adulthood with conduction system disease, LV dilatation, and VT (10). Moreover, the typical CMR finding in *LMNA/C*-DCM is the presence of linear mid-wall fibrosis in the basal interventricular septum, which is also a predilection site of CS and limits the diagnostic utility of CMR to differentiate between CS and *LMNA/C*-DCM (6, 11). The interaction between both diseases is conceivable, wherein the pathogenic mutation renders the myocardium susceptible to secondary damage. In particular, the *LMNA/C* variant presented in the first case is suspected to cause a nonsense-mediated mRNA decay (NMD), which prevents the expression of the truncated mutant protein, and has additional trans effects on the levels of lamin A protein expressed by the wild-type allele, decreasing its levels and causing a shift in the ratio of lamin A to lamin C (12). The release of proinflammatory cytokines such as TNF-α, IL-2, and Interferon-*γ* may accelerate disease deterioration in a susceptible mutation carrier. Interestingly, abnormal myocardial ^18^FDG uptake was previously observed in a patient with *LMNA/C* DCM, providing more evidences that inflammation may be a key factor in the development of *LMNA/C*-associated DCM (13).

Recurrent presyncope of unclear mechanism, ventricular fibrillation in ICD interrogation, and induced Brugada type-1 ECG were indicating a probable/definite diagnosis of BrS according to the Shanghai Score System (14). The VF episode is more suggestive of BrS, while a monomorphic VT resulting from scar-related re-entry is a typical finding in cardiac sarcoidosis (15, 16). Rarely, ventricular fibrillation and polymorphic VT are observed in CS, which can be explained by triggered activity resulting from early or delayed afterdepolarizations caused by increased intracellular calcium release (16). However, CS can imitate BrS due to extensive right ventricular involvement and inflammation. One theory regarding BrS suggests that the characteristic ECG pattern and the occurrence of ventricular arrhythmias may be attributed to the conduction slowing in the right ventricular outflow tract alongside with a delay in epicardial activation (17). Coronel et al. (18) demonstrated that conduction slowing in epicardial RVOT is associated with extensive regional fatty infiltration and fibrosis without evidence of associated mutations. A similar mechanism was suggested in ACM and could be the missing link between CS, ACM, and BrS phenocopies (19). The pronounced inflammatory involvement in this case, with evidence of edema and LGE in both the RV and LV, along with potential alterations in electrical properties, could potentially have led to a phenotypic overlap during the Ajmaline provocation test. On the other hand, the presence of CS could potentially impact the penetrance of an oligogenic BrS within the context of an overlap syndrome, stemming from an epigenetic effect induced by inflammation. BrS is considered an autosomal dominant disease, although alternative mechanisms of oligogenic inheritance have been proposed. Genetic studies have revealed that 30% of diagnosed BrS are associated with >350 rare variants in *SCN5A*, although almost 60% of these cases have negative results in genetic testing (20). The same *SCN5A* variant (p.C335R) has been linked to BrS, familial atrial fibrillation, and DCM (21). Since the same *SCN5A* variant can result in different phenotypes, it is reasonable to assume that additional factors contribute to the development of the corresponding phenotype, e.g., oligogenic, polygenic, epigenetic, or inflammatory mechanisms.

In the third case of suspected HCM, the CMR and ^18^FDG-PET imaging raised suspicion of CS due to the unexpected evidence of acute myocardial inflammation. A heterozygous *MYBPC3* variant (p.Arg1268Trp) of unclear significance and an allele count higher than expected for a pathogenic variant (ExAC 0.03%) were found, which has not previously been reported in *MYBPC3*-related cardiomyopathy (22). However, *MYBPC3* mutations are frequently associated with autosomal dominant DCM, NCCM, and RCM and are the most common cause of familial HCM (23). Notably, incomplete penetrance of the *MYBPC3* mutations is suggested (24). In this case, no pathological genetic variant was identified; instead, a VUS was found, leading us to hypothesize that CS might be associated with an enhancement of the penetrance and expression of a VUS within the context of an overlap syndrome, thereby leading to the HCM phenotype. As in the previous case, this could be attributed to an epigenetic effect induced by inflammation.

The overlap between cardiac sarcoidosis and ACM, as well as phenocopies of ACM caused by cardiac sarcoidosis, has been previously described, including a case from our group (25). CS represents one of the most common phenocopies of ARVC (26). Vasaiwala et al. (27) reported that 15% of initially diagnosed ACM cases were reclassified as CS based on invasive findings. The revised international Task Force Criteria (TFC) of 2010 lack reliability in differentiating between CS and ACM. Gasperetti et al. (26) distinguished ACM by noting larger right RVOT dimensions (RVOT dilation ≥ 35 mm) and T-wave inversions, while CS exhibited greater LV involvement, lower LVEF, and high-grade AVB. The positive ^18^FDG-PET results were exclusive to CS in this study. However, ACM may also exhibit a positive ^18^FDG-PET result during inflammatory phases, marking the transition from subclinical to overt disease phenotype (28–30). Both Protonotarios et al. and Smith et al. (29, 31) observed elevated myocardial FDG uptake in ACM patients, particularly in those with mutations in the desmoplakin gene. As a result, ACM may be misdiagnosed as CS, warranting a myocardial biopsy to clarify the diagnosis. In our case, it remains unclear whether CS was incidentally discovered or if the early death of the patient resulted from the combined unfavorable effects of CS and ACM due to the inability to perform ^18^FDG-PET scans, owing to the rapid and severe progression of the disease. However, based on the negative autopsy for sarcoid granulomas, we rather assume a coexistence in this case.

## Conclusion

Cardiac sarcoidosis and all types of inherited cardiomyopathies may interact in several different ways, changing the clinical presentation and likely the course of the disease. Overlapping pathologies are frequently overlooked and associated with an unfavorable clinical course. Delayed or incomplete diagnosis bears the risk of suboptimal treatment. ^18^FDG-PET, endomyocardial biopsies, and genetic evaluation should be considered to all patients with suspected inherited cardiomyopathy and/or cardiac sarcoidosis to obtain a clear diagnosis.

## Data Availability

The original contributions presented in the study are included in the article/Supplementary Material; further inquiries can be directed to the corresponding author.
