# Corrigendum: Case Report: Four cases of cardiac sarcoidosis in patients with inherited cardiomyopathy—a phenotypic overlap, co-existence of two rare cardiomyopathies or a second-hit disease

**DOI:** 10.3389/fcvm.2024.1372782

**Published:** 2024-02-14

**Authors:** Hans Ebbinghaus, Laura Ueberham, Daniela Husser-Bollmann, Andreas Bollmann, Ingo Paetsch, Cosima Jahnke, Ulrich Laufs, Borislav Dinov

**Affiliations:** ^1^ Department for Electrophysiology, Heart Center of Leipzig, Leipzig, Germany; ^2^ Klinik und Poliklinik für Kardiologie, Universitätsklinikum Leipzig, Leipzig, Germany; ^3^ Department of Cardiology, Medical University of Giessen, Giessen, Germany

**Keywords:** cardiac sarcoidosis, non-ischemic cardiomyopathy, familial cardiomyopathy, ventricular tachyarrhythmias, conduction disease

**A Corrigendum on**
Case Report: Four cases of cardiac sarcoidosis in patients with inherited cardiomyopathy—a phenotypic overlap, co-existence of two rare cardiomyopathies or a second-hit disease By Ebbinghaus H, Ueberham L, Husser-Bollmann D, Bollmann A, Laufs U and Dinov B (2023). Front. Cardiovasc. Med. 10:1328802. doi: 10.3389/fcvm.2023.1328802

## Error in author list

In the published article, there was an error in the author list, and authors Ingo Paetsch and Cosima Jahnke were erroneously excluded. The corrected author list appears below.

Hans Ebbinghaus^1^*, Laura Ueberham^2^, Daniela Husser-Bollmann^1^, Andreas Bollmann^1^, Ingo Paetsch^1^, Cosima Jahnke^1^, Ulrich Laufs^2^ and Borislav Dinov^3^

*Affiliations of all authors as they appear in the published original version of the article*

^1^Department for Electrophysiology, Heart Center of Leipzig, Leipzig, Germany, ^2^Klinik und Poliklinik für Kardiologie, Universitätsklinikum Leipzig, Leipzig, Germany, ^3^Department of Cardiology, Medical University of Giessen, Giessen, Germany

The authors apologize for this error and state that this does not change the scientific conclusions of the article in any way. The original article has been updated.

